# Maternal and Clinical Outcomes of Placenta Accreta Spectrum: Insights from a Retrospective Study in Bahrain

**DOI:** 10.3390/life15060978

**Published:** 2025-06-18

**Authors:** Kareeza Selby Chacko, Reem Satam AlSubeaei, Soumya Sunil Nair, Nusrat Khalil Kazi, Rafiea Jeddy

**Affiliations:** 1 School of Medicine, Royal College of Surgeons in Ireland-Bahrain (RCSI-Bahrain), Busaiteen P.O. Box 15503, Bahrain; 22202302@rcsi-mub.com (K.S.C.); 20200254@rcsi-mub.com (R.S.A.); 20205745@rcsi-mub.com (S.S.N.); 2 Department of Obstetrics and Gynecology, Salmaniya Medical Complex, Manama P.O. Box 12, Bahrain; nusrat_khalil_kazi@yahoo.com; 3 Department of Obstetrics and Gynecology, Royal College of Surgeons in Ireland-Bahrain (RCSI-Bahrain), Busaiteen P.O. Box 15503, Bahrain

**Keywords:** placenta accreta spectrum (PAS), placenta previa, cesarean delivery, maternal morbidity, obstetric complications

## Abstract

Placenta accreta spectrum (PAS) refers to a group of abnormal placental attachments in which the placenta adheres too deeply to the uterine wall, with varying degrees of invasion classified as accreta, increta, or percreta. Increased rates of uterine surgeries, advanced maternal age, and cesarean deliveries have all contributed to an increase in the incidence of PAS. Complications associated with PAS can lead to severe intrapartum or postpartum hemorrhage, hysterectomy, and significant maternal morbidity, making early diagnosis and management crucial for improving outcomes. Understanding the epidemiology and risk factors of PAS is crucial for developing early detection protocols and preventive strategies. Localized data, particularly from Bahrain, can inform targeted care approaches and optimize resource allocation, ultimately leading to improved clinical guidelines, enhanced patient education, and better healthcare outcomes for affected women. There are growing concerns about the impact of PAS on maternal health and healthcare resources in Bahrain, similar to trends observed in other regions. To improve patient education and management strategies, it is essential to comprehend the regional patterns, characteristics, and outcomes associated with PAS. However, the absence of comprehensive data specific to Bahrain hinders effective clinical decision-making and policy development. Addressing this gap is imperative for advancing maternal healthcare in the region.

## 1. Introduction

Placenta accreta spectrum (PAS) disorders encompass a continuum of abnormal placental invasions into the uterine wall, categorized into three main types: accreta, increta, and percreta. These disorders arise due to a disruption in the normal development of the decidua basalis, typically following prior uterine interventions, such as cesarean sections or curettage. In PAS, the placental villi anchor abnormally to the myometrium, with the extent of invasion distinguishing the subtypes—ranging from surface adherence in accreta to deep infiltration through the myometrium in increta and in percreta, beyond the uterine serosa, possibly affecting adjacent organs such as the bladder [1,2,3].

Once considered a rare obstetric complication, placenta accreta spectrum (PAS) has become increasingly prevalent in recent decades, a trend largely attributed to the rising rates of cesarean deliveries over the past fifty years. The current literature estimates the incidence of PAS to be approximately 1 in 1000 deliveries, with the reported prevalence ranging from 0.04% to as high as 0.9% across various populations. This trend closely parallels the increasing rates of cesarean deliveries and other uterine surgeries, such as myomectomy, endometrial ablation, or curettage. Studies have also identified maternal age over 35, multiparity, placenta previa, cesarean scars, and assisted reproductive technologies as important predisposing factors [4,5,6,7,8].

While studies on placenta accreta spectrum (PAS) disorders have been well-documented in the international literature, there remains a scarcity of regional data, particularly within the Gulf Cooperation Council (GCC) countries. However, emerging evidence from Qatar, Saudi Arabia, and the United Arab Emirates (UAE) has started to shed light on the rising burden of PAS, largely attributed to increasing cesarean section rates across the region [9,10,11,12].

Accurate and timely prenatal diagnosis of PAS is essential for optimal maternal and fetal outcomes. Ultrasound remains the first-line modality for screening and diagnosis, with features such as loss of the retroplacental clear zone, abnormal vascularity, and myometrial thinning serving as key indicators. However, when ultrasound findings are inconclusive or placental location is suboptimal, MRI can offer complementary details regarding the extent and depth of invasion, thereby aiding surgical planning [2,13,14,15].

In the context of Bahrain, cesarean section rates have steadily increased over the past two decades, reflecting a global trend toward more surgical deliveries. However, there is a paucity of local data describing the epidemiology, diagnosis, and outcomes of PAS in this population. This presents a significant gap in knowledge, particularly considering the implications of PAS for maternal health, surgical planning, and healthcare resource utilization in tertiary hospitals such as Salmaniya Medical Complex (SMC), Bahrain’s main referral center for high-risk obstetric cases.

This retrospective study aims to evaluate the incidence, clinical characteristics, diagnostic accuracy, and maternal outcomes associated with placenta accreta spectrum disorders—specifically placenta accreta and percreta—over the past four years in Bahrain. By analyzing data collected from Salmaniya Medical Complex, the study seeks to identify common risk factors, assess current diagnostic practices, and evaluate maternal and surgical outcomes, thereby contributing to the development of more effective clinical guidelines and improving patient care strategies in the region.

## 2. Materials and Methods

This retrospective observational study was conducted at Salmaniya Medical Complex, a tertiary care center in Bahrain, over a four-year period from January 2021 to December 2024. Ethical approval was obtained from the institutional review board prior to data collection.

### 2.1. Case Identification and Inclusion Criteria

All confirmed cases of placenta accreta spectrum (PAS)—including placenta accreta, increta, and percreta—were identified through a comprehensive review of patient records, including operative notes and histopathology reports. Only cases with clearly documented intraoperative findings or histopathological confirmation of PAS were included. Cases lacking definitive evidence of PAS were excluded.

To ensure data accuracy, all patient records were individually cross-checked against our institutional database to confirm Placenta Accreta Spectrum (PAS) diagnoses.

To mitigate misclassification bias, two independent reviewers verified all PAS diagnoses using predefined criteria (e.g., histopathological confirmation or intraoperative findings). Discrepancies were resolved through adjudication by a third senior clinician.

### 2.2. Data Collection

Relevant maternal, obstetric, surgical, and neonatal data were manually extracted from hospital records using a standardized electronic data collection template.

The information gathered included maternal age, nationality, gravidity, parity, gestational age at delivery, prior cesarean sections, history of uterine surgery, comorbid conditions (e.g., G6PD deficiency, sickle cell disease, infertility treatments), and the presence of placenta previa.

Details regarding PAS classification (accreta, increta, percreta), delivery method, estimated blood loss, transfusion requirements, need for hysterectomy, surgical complications, ICU admission, and maternal morbidity were recorded. Neonatal outcomes, such as birth weight, APGAR scores, gestational age at birth, and NICU admissions, were also documented.

### 2.3. Data Analysis

Data were analyzed descriptively. Frequencies and percentages were used to summarize categorical variables, while means, medians, and ranges were used for continuous variables. Data tabulation and simple statistical summaries were performed manually and using spreadsheet software (Microsoft Excel). Inferential statistical tests were performed using SPSS Version 29 to analyze trends, patient characteristics, and outcomes associated with PAS.

## 3. Results

During the study period, among 30,004 deliveries recorded at Salmaniya Medical Complex, 20 (<1%) cases of PAS disorders were diagnosed. Placenta accreta, placenta percreta and placenta increta were confirmed by histopathologic analysis among 11 (55%), 6 (30%) and 3 (15%). Placenta previa was diagnosed among 12 (60%) of the patients.

### 3.1. Incidence and Demographic Characteristics

Over the four-year study period, 20 cases of PAS were identified. The annual distribution of cases demonstrated an increasing trend, with 3 cases in 2021, 2 in 2022, 4 in 2023, and 11 in 2024. Potential contributing factors to the observed increase in cases may include a genuine rise in the prevalence of Placenta Accreta Spectrum (PAS), heightened awareness among clinicians regarding this condition, and changes in referral patterns within the healthcare system. Further investigation, employing a larger and prospectively collected dataset, is necessary to definitively determine whether this increase represents a true rise in PAS incidence or is attributable to other factors influencing case identification.

The mean maternal age at delivery was 35.35 years (SD = 4.36 years). The majority of patients were Bahraini nationals, representing 65% of the cohort (13/20), reflecting the demographic composition of the obstetric population served by the institution.

### 3.2. Risk Factors and Clinical Presentation

A detailed assessment of obstetric risk factors revealed that placenta previa was a prominent finding, documented in 60% of cases (12/20). A history of prior cesarean delivery was also highly prevalent, observed in 85% of the study population (17/20). Other notable risk factors included two cases of GDM on T. Glucophage, G6PD deficiency in 10% (2/20) of cases, thalassemia B trait in one case, one case of a patient with COVID-19, and three cases of APH. These findings underscore the importance of considering these factors in risk stratification for PAS.

Gestational age at delivery ranged from 28 to 38+ weeks. The majority of deliveries occurred at term (≥37 weeks), accounting for 45% of cases (9/20), while 55% (11/20) were classified as preterm.

Based on intraoperative findings and histopathological confirmation, the distribution of PAS subtypes was as follows: placenta accreta was identified in 11 (55%), placenta increta in 3 (15%), and placenta percreta in 6 (30%) patients.

### 3.3. Maternal Outcomes

Maternal morbidity was significant in this cohort. As shown in Table 1, Postpartum hemorrhage (PPH) occurred in 85% of patients (17/20). The median estimated blood loss (EBL) was 4 L (range: 0.5–10 L), with 70% (14/20) experiencing severe hemorrhage exceeding 2 L. The majority of patients with significant blood loss required blood transfusion, with a median of 4 units of packed red blood cells (range: 1–12 units) and 5.5 units of fresh frozen plasma (range: 2–8 units) administered. In addition, platelets or cryoprecipitate were required in 65% of cases (13/20), indicating the severity of coagulopathy in some patients.

Hysterectomy was performed in 65% of patients (13/20), with 50% (10/20) undergoing elective cesarean hysterectomy and 15% (3/20) requiring emergency procedures due to uncontrolled hemorrhage or surgical complications. Bladder injury occurred in 15% of cases (3/20) during hysterectomy, necessitating urological intervention and highlighting the risk of adjacent organ damage in severe PAS. Postoperative intensive care unit (ICU) admission was required in 20% of patients (4/20) for close monitoring and management of complications. There were no maternal deaths in this period.

### 3.4. Neonatal Outcomes

Neonatal outcomes were also impacted. The median birth weight was 2.69 kg (range: 0.435–4.39 kg), with 25% of neonates (5/20) classified as low birth weight (<2.5 kg). APGAR scores were generally favorable, with a median score of 9 at 5 min and 10 at 10 min. Preterm birth occurred in 55% of cases (11/20). Key neonatal outcomes, such as preterm birth, birth weight, and early APGAR scores, are presented in Table 2.

### 3.5. Management Strategies

A variety of management strategies were employed. Elective cesarean hysterectomy was the primary surgical approach in 50% (10/20) of cases, while 15% (3/20) underwent emergency hysterectomy due to intraoperative complications. Conservative management techniques, including Bakri balloon placement or uterine packing, were attempted in 25% of cases.

The management of PAS often required a multidisciplinary approach [16]. Prophylactic ureteric stenting was performed in 15% of patients (3/20) with the intention to minimize the risk of ureteral injury during surgery; however, recent studies have questioned the efficacy of the use of ureteral stents [17,18]. Intraoperative management of bladder injuries required urological expertise in 5% of cases (1/20), emphasizing the importance of a coordinated team approach.

### 3.6. Inferential Analysis

Several statistically significant associations were identified in the study. Patients diagnosed with placenta previa exhibited significantly higher odds of undergoing hysterectomy compared to those without placenta previa (*p* = 0.0004). Placenta previa was also significantly associated with an increased risk of preterm delivery (*p* = 0.0014).

Blood loss varied significantly across different PAS subtypes, with cases of placenta percreta demonstrating markedly higher estimated blood loss than those with accreta or increta (ANOVA *p* < 0.0001). Additionally, patients who experienced postpartum hemorrhage (PPH) had significantly greater blood loss compared to those without PPH (*p* = 0.0005).

A significant difference in mean birth weight was observed among PAS subtypes (*p* = 0.0002), with preterm neonates having significantly lower birth weights than those delivered at term (*p* = 0.00013). While the relationship between PAS subtype and low birth weight did not reach statistical significance, it approached significance (*p* = 0.065). No statistically significant association was found between PAS subtype and the need for ICU admission (*p* = 0.129).

## 4. Discussion

This retrospective analysis was conducted at Salmaniya Medical Complex, Bahrain’s primary tertiary referral center for obstetric care and the sole facility managing all cases of Placenta Accreta Spectrum (PAS) in the country. Notably, this is the only study of its kind conducted in Bahrain. The study period spanned from 1 January 2021 to 31 December 2024, during which 20 confirmed cases of PAS were identified and analyzed. This research highlights significant trends and challenges in managing this increasingly prevalent obstetric complication.

A notable finding was the apparent increase in PAS incidence during the study period, with a marked rise in cases observed in 2024. While this trend could be attributed to enhanced diagnostic accuracy resulting from an increased clinical awareness and the utilization of advanced imaging modalities, such as MRI and ultrasound [14,15], the inconsistent documentation of these diagnostic methods within our dataset precludes a definitive conclusion. It is also plausible that this increase reflects a genuine rise in PAS prevalence within our population, potentially mirroring global trends associated with escalating rates of cesarean delivery and advanced maternal age [19]. Further prospective studies are warranted to elucidate the underlying factors driving this observed increase and to establish accurate incidence rates within the region.

Placenta previa and a history of prior cesarean delivery emerged as prominent risk factors in our cohort, present in 60% and 85% of cases, respectively. The established association between these factors and PAS is likely mediated by uterine scarring and aberrant placental implantation at the site of previous uterine incisions [20]. While less prevalent, other risk factors, such as G6PD deficiency (10%) and thalassemia b trait (5%), were also observed, suggesting a potential role for these conditions in the pathogenesis of PAS, although further investigation is needed to confirm these associations. Future case-control studies could assess whether these hematologic disorders influence placental adherence, vascular remodeling or healing of uterine scars, potentially offering novel insights into PAS pathophysiology in specific populations.

The significant maternal morbidity observed in our study, characterized by a high rate of postpartum hemorrhage (85%) and a substantial proportion of patients requiring hysterectomy (65%), underscores the clinical challenges posed by PAS. The median estimated blood loss of 4 L, with nearly 70% of patients experiencing severe hemorrhage exceeding 2 L, highlights the potential for life-threatening complications [21]. The hysterectomy rate, while consistent with some reports [22], was somewhat higher than others [23], possibly reflecting variations in surgical management strategies and the severity of PAS cases encountered at our referral center. The occurrence of bladder injury in 15% of cases further emphasizes the importance of meticulous surgical technique and multidisciplinary collaboration to minimize iatrogenic complications, with recent studies demonstrating improved diagnostic accuracy for detecting bladder involvement in PAS through the combined use of ultrasound and MRI [24].

This study also incorporated inferential statistics to assess the relationships between key risk factors and clinical outcomes. Notably, placenta previa was significantly associated with both increased hysterectomy rates and a higher risk of preterm delivery. Furthermore, blood loss was significantly higher among patients with percreta and in those who developed postpartum hemorrhage. These findings reinforce the clinical importance of antenatal risk stratification. Lower birth weight was observed in preterm deliveries and was significantly influenced by PAS subtype, although not categorically associated with low-birth-weight classification. These inferential insights underscore the complex interplay between placental pathology and obstetric outcomes, and emphasize the need for early diagnosis and planned multidisciplinary care.

In terms of neonatal outcomes, our study revealed a notable proportion of preterm births (55%) and low-birth-weight infants (25%), suggesting a potential impact of PAS on fetal well-being. These findings are consistent with the known association between PAS and planned preterm delivery to mitigate maternal risks [25]. However, the generally favorable APGAR scores observed in our cohort indicate that, with appropriate management, adverse neonatal outcomes can be minimized.

The management of PAS in our cohort involved a range of strategies, including elective cesarean hysterectomy, conservative management techniques, and multidisciplinary interventions. The decision to pursue elective cesarean hysterectomy was often driven by the severity of placental invasion and the presence of significant risk factors [26,27]. This approach is supported by recent cost-effectiveness analyses demonstrating that planned hysterectomy offers better maternal outcomes and resource efficiency [28,29]. Additionally, professional society guidelines now increasingly recommend planned cesarean hysterectomy in cases of confirmed placenta accreta spectrum due to its association with improved surgical outcomes [30].

While conservative management, including Bakri balloon placement and uterine packing [31], was attempted in some cases, the limited data on the success rates of these approaches in our cohort preclude a definitive assessment of their efficacy; nonetheless, studies have demonstrated that such conservative approaches can significantly reduce blood loss and transfusion needs, with fewer genitourinary injuries compared to hysterectomy [23,32,33]. These findings underscore the need for individualized management plans tailored to the specific clinical presentation and the expertise of the multidisciplinary team. Moreover, the use of interventional radiology, such as balloon occlusion or embolization of pelvic vessels, has emerged as a valuable adjunct to conservative management in selected cases, although availability and expertise remain limiting factors [34]. These findings underscore the need for individualized management plans tailored to the specific clinical presentation and the expertise of the multidisciplinary team [35].

### 4.1. Limitations

Several limitations warrant consideration when interpreting the findings of this study. The retrospective design inherently introduces the potential for selection bias and the possibility of incomplete data capture. While we made every effort to ensure accuracy, the reliance on handwritten birth registers and medical notes presented some challenges during data extraction. The legibility and completeness of these handwritten records varied, which required careful and time-consuming reviewing to ensure the most accurate data abstraction possible. The lack of consistent documentation regarding diagnostic modalities and PAS subtype classification also limits the scope of our analysis. Moreover, the relatively small sample size and single-center nature of the study suggest caution when generalizing our findings to other populations.

### 4.2. Future Studies

Future prospective, multi-center studies with larger sample sizes would be valuable to confirm and expand upon these initial observations, and to further elucidate the complex interplay of risk factors, diagnostic strategies, and management approaches in PAS. Such studies should also focus on assessing long-term maternal and neonatal outcomes to inform evidence-based clinical practice guidelines.

## 5. Conclusions

This retrospective study is the first of its kind conducted in Bahrain and offers valuable insights into the incidence, risk factors, management, and outcomes of placenta accreta syndrome (PAS) in the region. The observed increase in PAS cases, along with the significant maternal morbidity associated with this condition, highlights the need for heightened clinical awareness, improved diagnostic strategies, and optimized multidisciplinary management protocols. Given that placenta previa and prior cesarean delivery are prominent risk factors, targeted screening and counseling efforts for women with these characteristics are essential. While elective cesarean hysterectomy is a common management approach, it is crucial to develop individualized treatment plans that take into account the severity of PAS and patient preferences. Further prospective, multi-center research is needed to validate these findings, evaluate the effectiveness of different management strategies, and ultimately enhance maternal and neonatal outcomes for women affected by PAS. Incorporating these findings into local clinical guidelines and healthcare policies will significantly improve patient care and resource allocation in Bahrain.

## Figures and Tables

**Table 1:** Maternal outcomes and complications (n = 20).

Outcome	Value (n = 20)
**Postpartum hemorrhage (PPH)**	85% (17/20)
**Median blood loss (L)**	4 L (Range: 0.2–10)
**Severe hemorrhage (>2 L)**	70% (14/20)
**Blood transfusion**	
**-** Packed RBCs (median units)	4 (Range: 1–12)
**-** FFP (median units)	5.5 (Range: 2–8)
**Hysterectomy**	65% (13/20)
**-** Elective	50% (10/20)
**-** Emergency	15% (3/20)
**Bladder injury**	15% (3/20)
**ICU admission**	20% (4/20)

**Table 2:** Neonatal outcomes (n = 20).

Outcome	Value (n = 20)
**Preterm birth (<37 weeks**)	55% (11/20)
**Mean birth weight (kg)**	2.69 (Range: 0.435–4.39)
**Low birth weight (2.5 kg)**	25% (5/20)
**APGAR < 7 at 5 min**	15% (3/20)

## Data Availability

The data used in this study are not publicly available due to privacy and ethical restrictions. Access to the anonymized patient records is restricted by the hospital’s research ethics committee.
